# MiR-144-3p Enhances Cardiac Fibrosis After Myocardial Infarction by Targeting *PTEN*

**DOI:** 10.3389/fcell.2019.00249

**Published:** 2019-10-29

**Authors:** Xiaolong Yuan, Jinchun Pan, Lijuan Wen, Baoyong Gong, Jiaqi Li, Hongbin Gao, Weijiang Tan, Shi Liang, Hao Zhang, Xilong Wang

**Affiliations:** ^1^Guangdong Provincial Key Laboratory of Laboratory Animals, Guangdong Laboratory Animals Monitoring Institute, Guangzhou, China; ^2^National Engineering Research Center for Swine Breeding Industry, Guangdong Provincial Key Lab of Agro-Animal Genomics and Molecular Breeding, College of Animal Science, South China Agricultural University, Guangzhou, China

**Keywords:** miR-144-3p, *PTEN*, cardiac fibrosis, myocardial infarction, extracellular matrix

## Abstract

Myocardial infarction (MI) may cause heart failure and seriously harm human health. During the genesis of cardiac fibrosis after MI, the proliferation and migration of cardiac fibroblasts contribute to secretion and maintenance of extracellular matrix (ECM) components. Many miRNAs have been highly implicated in the processes of cardiac fibrosis after MI. However, the molecular mechanisms for how miRNAs involve in cardiac fibrosis remain largely unexplored. Based on MI model in miniature pigs, the potential miRNAs involved in MI were identified by using small RNA sequencing. Using human cardiac fibroblasts (HCFs) as a cellular model, EdU, Transwell, and the expression of ECM-related proteins were applied to investigate the cell proliferation, migration and collagen synthesis. In this study, using MI model based on miniature pigs, 84 miRNAs were identified as the differentially expressed miRNAs between MI and control group, and miR-144-3p, one of differentially expressed miRNAs, was identified to be higher expressed in infarct area. The cell proliferation, migration activity, and the mRNA and protein levels of the ECM-related genes were significantly increased by miR-144-3p mimic but significantly decreased by miR-144-3p inhibitor in cardiac fibroblasts. Furthermore, miR-144-3p was observed to repress transcription and translation of *PTEN*, and interfering with the expression of *PTEN* up-regulated the mRNAs and proteins levels of α*-SMA*, *Col1A1*, and *Col3A1*, and promoted the proliferation and migration of cardiac fibroblasts, which was in line with that of miR-144-3p mimics, but this observation could be reversed by miR-144-3p inhibitor. Collectively, miR-144-3p promotes cell proliferation, migration, and collagen production by targeting *PTEN* in cardiac fibroblasts, suggesting that miR-144-3p-mediated-PTEN regulation might be a novel therapeutic target for cardiac fibrosis after MI.

## Introduction

The myocardial infarction (MI) caused by coronary artery acute or persistent hypoxia-ischemia is a type of cardiovascular disease (Acharya, 2018), which may cause heart failure and seriously harm human health. Much evidence has suggested that MI accounts for approximate 7.6% of the total deaths in China in 2011 (Wang et al., 2017). Currently, it is estimated that there are approximate 2.5 million MI patients in China (Wang et al., 2017) and one million MI patients in Americans (Benjamin et al., 2017). The cardiac fibrosis is the pivotal pathological characteristic after MI, which causes heart remodeling and heart failure (Furtado et al., 2016; Kurose and Mangmool, 2016). A great deal of studies has demonstrated that cardiac fibrosis after MI is activated with the abnormal accumulation and collagen secretion of extracellular matrix (ECM) protein in the infarct area (Zamilpa and Lindsey, 2010; van Nieuwenhoven and Turner, 2013; Lu et al., 2015). During the processes of cardiac fibrosis, MI induces the proliferation of fibroblasts, which account for about 70% of cells in the healthy heart (Zak, 1974; Eghbali et al., 1989), to migrate to the injured myocardial site and differentiate to myofibroblasts to deposit fibrillar collagens such as types I (Col1A1) and III (Col3A1) (Shinde and Frangogiannis, 2014; Chistiakov et al., 2016). The synthesis and accumulation of collagens are the major features during cardiac fibrosis after MI (Brown et al., 2005; Porter and Turner, 2009). Moreover, the excessive ECM deposition not only leads to ventricular dilation, infarct expansion, and cardiac remodeling, but also causes changes in myocardial structure, function, and phenotype (Zamilpa and Lindsey, 2010). Although MI is critical danger to humans, the molecular mechanism for cardiac fibrosis and ECM deposition after MI remains unclear.

MicroRNAs (miRNAs), a kind of endogenous single non-coding RNAs with 20–23 nucleotides in length, have been suggested to play an critical role during cardiac fibrosis after MI (Chen et al., 2015; Yao et al., 2015; Zhu et al., 2016; Zhang and Cui, 2018). For example, miR-26a induces the protein expressions of α-smooth muscle actin (α-SMA), which is critical for fibroblasts differentiation to myofibroblasts (Willems et al., 1994), as well as the expressions of p-AKT, MMP9, and Col1A1 during cardiac fibrosis in rats (Zhang and Cui, 2018). MiR-21 promotes cardiac fibrosis via STAT3 signaling pathway in rats (Cao et al., 2017), and miR-101a mitigates interstitial fibrosis and has been recommended as the therapeutic potential for MI (Pan et al., 2012). miR-379 has been suggested as a novel biomarker for diagnosis of MI (Yi and An, 2018). Although these results provide novel therapeutic approaches for cardiac fibrosis after MI, the molecular mechanisms for how miRNAs involve in cardiac fibrosis remain unclear.

Previous studies have suggested that pigs are the valuable MI model (Gabisonia et al., 2019; Lim, 2019) due to the similar physiological and genomic characteristics to humans (Groenen et al., 2012). In this study, MI model was firstly established on miniature pigs to explore the potential miRNAs involved in MI by using small RNA sequencing. Then miR-144-3p, which had been reported to be significantly associated with MI in humans (Hu et al., 2014), was identified to express higher in infarct area in this study and was selected for further investigation to explore its performance on the proliferation, migration, and ECM synthesis of cardiac fibroblast cells. Moreover, the phosphatase and tensin homology gene (*PTEN)*, which had been suggested to participate in the occurrence of cardiac fibrosis after MI (Tao et al., 2017; Zhang and Cui, 2018), was determined as a target of miR-144-3p, and the biological functions of miR-144-3p-mediated-PTEN were further characterized on proliferation, migration, and ECM synthesis of cardiac fibroblast cells. These works provide new insights into discovering molecular mechanisms for cardiac fibrosis after MI.

## Materials and Methods

### Ethics Statement

All experiments in the present study were performed in accordance with the guidelines of the Animal Care and Use Committee of Guangdong Provincial Key Laboratory for Laboratory Animals and Guangdong Laboratory Animals Monitoring Institute.

### Creation of MI Model in Pigs

A total of young male Juema minipigs (Chen et al., 2016) weighting 20–25 kg were used to create MI model according to previous studies (Gabisonia et al., 2019; Lopez et al., 2019). Briefly, these pigs were raised in Guangdong Provincial Key Laboratory for Laboratory Animals, and this laboratory has been identified and recognized by Association for Assessment and Accreditation of Laboratory Animal Care International. These anesthetized pigs were randomly divided into sham operation control group (*n* = 3) and MI group (*n* = 3). After supine bound, these pigs were transected 7–10 cm in the left third intercostal space to expose the heart. Three MI pigs were created by permanent ligation of the trunk near one third of the apex after the first branch. The thoracic cavity was opened, and sutures were placed in the approximate position without ligation for the other three pigs of sham operation control group. BeneViewT5 and EDAN H100 were used to monitor the basic vital signs of animals. The success of ligation was judged and elevated by ST segment of electrocardiogram. After 4 weeks following the surgery, the myocardial infarcted areas of MI group and the corresponding areas of control group were collected and stored into liquid nitrogen soon for further suing.

### Library Constructions and Data Analysis of Small RNA Sequencing

The small RNA library constructions and sequencing services were provided by Genedenovo Biotechnology Co., Ltd. (Guangzhou, China) according to previous studies (Hafner et al., 2008; Reddy et al., 2009). Briefly, the total RNAs of infarct area in MI pigs and the same area in control pigs were extracted by TRIzol, and the RNA molecules in a size range of 18–30 nt were enriched by polyacrylamide gel electrophoresis. Then the 3′ and 5′ adapters were added and ligated to the RNAs. The ligation products were reversely transcribed by polymerase chain reaction (PCR) amplification, and 140–160 bp size PCR products were enriched to generate a cDNA library sequenced using Illumina Hiseq^TM^2500.

After sequencing, raw reads were filtered to generate the clean reads, including removing reads with low quality, without 3′ adapters, containing 5′ adapters, shorter than 18 nt or containing ployA. The clean reads were aligned with small RNAs in GenBank (Release 209.0) and Rfam (Burge et al., 2013) (Release 11.0) database to remove rRNA, scRNA, snoRNA, snRNA, and tRNA. Then the data were aligned with the pig reference genome (Sscrofa 11.1). All of the clean reads were searched in miRBase database (Griffiths-Jones, 2006) (Release 21) to identify known miRNAs, and the novel miRNAs were predicted by Mireap_v0.2^1^ with default parameters. The expression levels of miRNAs were calculated and normalized to transcripts per million.

### Cell Culture

The human cardiac fibroblasts (HCFs) (catalog no. 6300) were purchased from Sciencell Research Laboratories (Carlsbad, CA, United States), were cultured in fibroblast medium-2 (FM-2) which is a complete medium designed for optimal growth of normal HCFs *in vitro* (Sciencell), and were incubated at 37°C in 5% CO_2_. Cells were passaged when the cell confluence achieved 80–90%, and 3rd or 4th passages of HCFs were used for following experiments.

Human cardiac fibroblasts were seeded and cultured into six-well plate. When cells reached 70% coverage of one well, miR-144-3p mimics (50 nM), miR-144-3p mimic control (50 nM), miR-144-3p inhibitors (150 nM), miR-144-3p inhibitor control or PTEN-specific siRNAs (150 nM) (RiboBio, Guangzhou, China) was transfected into cells using Lipofectamine^TM^ 3000 Reagent (Invitrogen, Carlsbad, CA, United States) in antibiotic-free medium. The transfected cells were incubated at 37°C for 24 h and then were replaced with the fresh complete medium. Cells were maintained in culture until other experiments.

### Quantitative Real-Time Polymerase Chain Reaction (qRT-PCR)

For mRNA and miRNA expression analysis, the total RNA was extracted from HCFs by using TRIzol reagent (Invitrogen, United States) according to the manufacturers protocol. The quantity of RNA was assessed spectrophotometrically using a NanoDrop One (NanoDrop Technologies, Thermo, United States). Then 0.5 μg of total RNA was reverse transcribed into cDNA using Reverse TransScript Kit (Toyobo, Takara, Japan). The mRNA expressions were performed with real-time PCR by using Maxima SYBR Green qRT-PCR Master Mix Kit (Takara, Japan) with *GAPDH* as the internal control in a LightCycler Real-Time PCR system. The relative expression of miR-144-3p was detected using THUNDERBIRD SYBR qPCR Kit (Toyobo, Japan) with U6 as the internal control in a LightCycler Real-Time PCR system. The relative gene expression levels were calculated based on the 2^–△^^△^^ct^ method. All procedures were repeated in at least triplicate. The primer sequences of qRT-PCR are shown in Supplementary Table S1.

### Proliferation Assay of HCFs

EdU cell proliferation kit (RiboBio, Guangzhou, China) was used to measure HCFs proliferation according to the manufacturer’s instructions. HCFs were transfected with miR-144-3p mimics (50 nM), miR-144-3p mimic control (50 nM), miR-144-3p inhibitors (150 nM), miR-144-3p inhibitor control or PTEN-specific siRNAs (150 nM) incubation 24 h. After 24 h incubation, the HCFs were treated with EdU (20 μM) for 2 h at 37°C, following fixation with 4% paraformaldehyde, permeabilizing treatment with 0.5% Triton X-100, and staining with Apollo^®^567 and Hoechst 33342. The photographs of cells were taken using a fluorescent microscope (Nikon, Japan).

### Transwell Migration Assay of HCFs

The transwell chamber (8 μm pore size, Corning, United States) were used to examine the HCFs migration ability. After transfection treatments, the HCFs were digested with 0.25% trypsin (Hyclone, United States) and re-suspended with FM-2 without fetal bovine serum (FBS). The 0.6 mL of FM-2 with 0.5% FBS was added into lower chamber. Then 100 μL of cell suspension solution was added into the upper chambers and incubated with 5% CO_2_ atmosphere at 37°C for 12 h. After removing the medium, 1 × PBS was used to wash the migrated cells on the both side of the membranes and fix the cells with 4% glutaraldehyde for 20 min. Then the unmigrated cells on the upper side of the membrane were removed with cotton swab. After that, the cells on the bottom side of the membranes were stained with crystal violet for 30 min, and the number of the cells was counted with microscope (Nikon, Japan) after washing by 1 × PBS. The cells that had migrated through the membrane were stained and counted.

### Western Blotting

Total protein was isolated from the HCFs using the total protein kit (Applygen, China). Then the protein was determined using the bicinchoninic acid protein assay kit (Thermo Fisher Scientific, United States). The primary antibodies were α-SMA (Absin, China), Col1A1 (Absin, China), Col3A1 (Absin, China), and *PTEN* (Absin, China) with GAPDH (Abcam, United Kingdom) serving as an internal control. Goat anti-rabbit IgG-HRP was used as a secondary antibody (Santa Cruz, United States). 80 μl proteins taken out with a pipette were electrophoresed on SDS-PAGE and transferred onto polyvinylidene difluoride membranes (PVDF, Millipore, MA, United States). The membranes were blocked with 5% non-fat milk in PBS containing a percentage of Tween-20 for 1.5 h and then incubated overnight at 4°C with α-SMA (1:2000, Rabbit), Col1A1 (1:1000, Rabbit), Col13A1 (1:1000, Rabbit), PTEN (1:1000, Rabbit), and GAPDH (1:10000, Rabbit) followed with secondary antibodies for 1 h. All proteins were visualized with the ECL-chemiluminescent kit (Sigma, United States), and the quantification was performed using densitometry with Image J software.

### Luciferase Reporter Assay

The mature sequences of miR-144-3p, located at chr17:28861533-28861618 (-), were obtained from miRBase^2^. The putative target genes for miR-144-3p were predicted and overlapped by three algorithms: TargetScan^3^ (v7.0), miRanda^4^ (v3.3a), and RNAhybrid^5^ (v2.1.2). To generate the reporter vectors containing the potential binding sites of miR-144-3p, the DNA fragments of the 3′UTR of target gene *PTEN* (NCBI Accession Gene ID: 5728), were amplified from the extracted DNA of HCFs and then cloned into the pmirGLO luciferase reporter plasmid (Promega, United States). Two constructs of pmirGLO luciferase reporter plasmid were generated: MUT-PTEN (with mutation of part of miR-144-3p binding site sequence) and WT-PTEN (containing the wild-type miR-144-3p binding site sequence). 500 ng of the pmirGLO luciferase reporter plasmid and appropriate miRNA plasmid were co-transfected with lipofectamine 3000 (Invitrogen, United States) into HCFs. 24 h after transfection, the luciferase expression was determined using the Dual-Glo^TM^ Luciferase Reporter Assay Kit (Promega, United States) according to the manufacturer’s protocol. The pRL-TK vector (Promega, United States) containing Renilla luciferase was also co-transfected for normalization in all relevant experiments.

### Statistical Analyses

The differentially expressed miRNAs of small RNA sequencing were identified by using edgeR (Robinson et al., 2010) with *P* < 0.05. The potential target genes of the differentially expressed miRNA were predicted by three algorithms: TargetScan (v7.0), miRanda (v3.3a), and RNAhybrid (v2.1.2). The Gene Ontology (GO) enrichment analysis were conducted by WEB-based Gene Set Analysis Toolkit^6^. The KEGG pathway enrichment analysis were undertaken by R package “clusterProfiler”(Yu et al., 2012). Data in this research were expressed as mean ± standard deviation (SD). The significant mean differences were evaluated by student’s *t*-test for two groups and by one-way ANOVA analysis for more than two groups. ^∗^ indicates *P* < 0.05; ^∗∗^ indicates *P* < 0.01.

## Results

### Differentially Expressed miRNAs Between MI and Control Pigs

**FIGURE 1:**
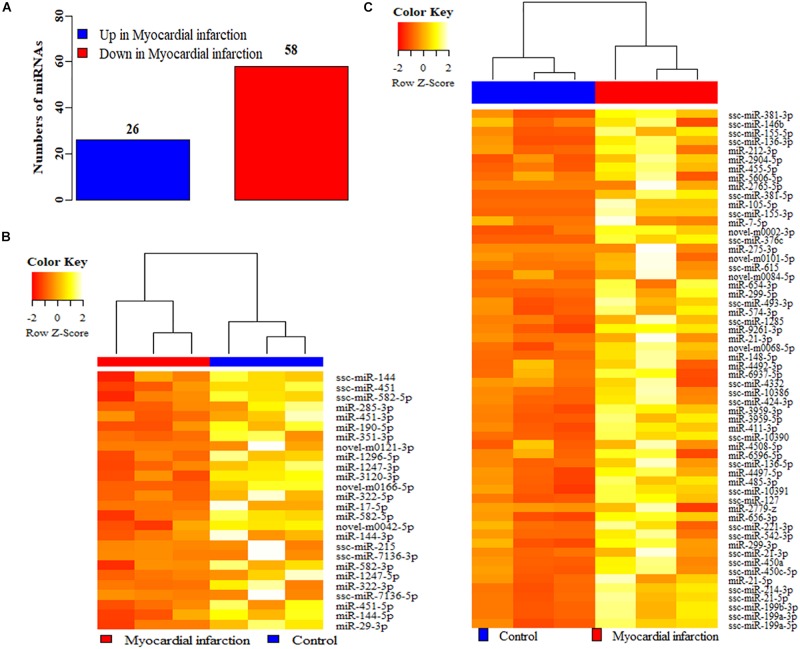
The differentially expressed miRNAs between MI and control groups. **(A)** The differentially expressed miRNAs between MI and control groups. **(B)** The cluster analysis of the up-regulated **(B)** and down-regulated **(C)** miRNAs in MI, compared to control groups. MI: myocardial infarction.

The genome wide miRNA profiles of the infarct area in MI pigs and the corresponding areas in control pigs were acquired by small RNA sequencing. The summary counts for the small RNA sequencing were tabled in the Supplementary Table S2. After data analysis, 1547 miRNAs were detected between MI and control group, and 84 miRNAs were identified as the differentially expressed miRNAs (Figure 1), including 26 up-regulated miRNAs and 58 down-regulated in MI (Figure 1A), compared to control group (Figure 1, see Supplementary Table S3). Moreover, both 26 up-regulated (Figure 1B) and 58 down-regulated (Figure 1C) miRNAs were clearly clustered into two groups: MI and control group. The GO terms of potential target genes of these differentially expressed miRNAs were mostly enriched in biological regulation, cellular process, ECM, organelle and catalytic activity (see Supplementary Figure S1), and the mostly enriched KEGG pathways were Axon guidance, PI3K-Akt signaling pathway, MAPK signaling pathway, and signaling pathways regulating pluripotency of stem cells (see Supplementary Figure S2).

### miR-144-3p Promotes Proliferation and Migration of Cardiac Fibroblasts

**FIGURE 2:**
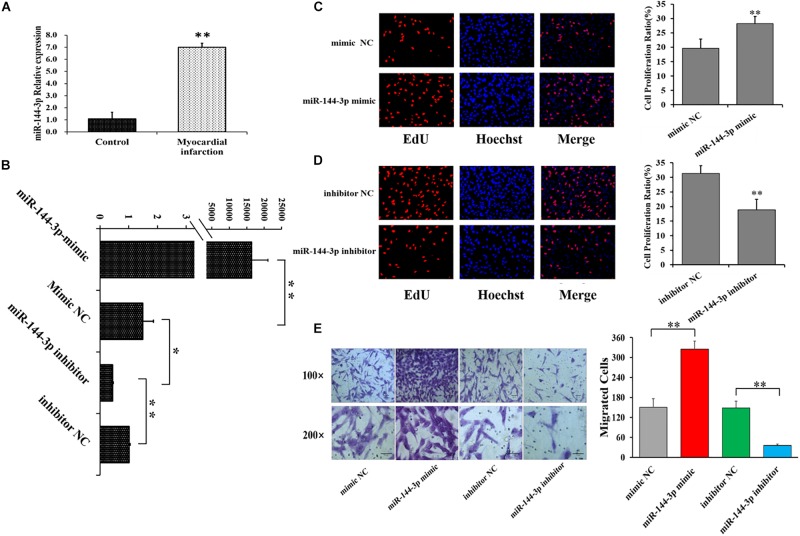
miR-144-3p promotes cell proliferation and migration in human cardiac fibroblasts (HCFs). **(A)** The expression of miR-144-3p in MI and control groups by qRT-PCR. **(B)** miR-144-3p mimic increased while miR-144-3p inhibitors decreased the expression of miR-144-3p in HCFs. Effect of miR-144-3p mimic **(C)** and inhibitor **(D)** on the proliferation of HCFs using EdU staining. **(E)** Effect of miR-144-3p on the migration of HCFs. Bars: 100 μm for 100 × figures, and 50 μm for 200 × figures. *N* ≥ 3. ^∗^*P* < 0.05, ^∗∗^*P* < 0.01. Data are shown as mean ± SD.

In the present study, miR-144-3p was identified as one of the differentially expressed miRNAs by small RNA sequencing (Figure 1B and Supplementary Table S3), and miR-144-3p was further validated to be higher expressed in MI area by qRT-PCR, compared to control pigs (Figure 2A). These observations implicated the potential role of miR-144-3p in cardiac fibrosis after MI. To further explore the role of miR-144-3p in cardiac fibrosis, the oligonucleotide mimics or inhibitors of miR-144-3p were constructed and transfected into HCF cells (Figure 2B). qRT-PCR assays confirmed that miR-144-3p mimic markedly increased the expression of miR-144-3p (Figure 2B), and miR-144-3p inhibitor significantly decreased the expression of miR-144-3p (Figure 2B). Compared to control group (NC), the cell proliferation ratio of HCFs was significantly increased by miR-144-3p mimic (Figure 2C) but significantly decreased by miR-144-3p inhibitor (Figure 2D). Moreover, miR-144-3p mimic was observed to significantly increase the migration activity of HCFs (Figure 2E), but miR-144-3p inhibitors significantly decreased migration activity of HCFs (Figure 2E). These results suggested that miR-144-3p could promote the cell proliferation and migration in HCFs.

### miR-144-3p Promotes Collagen Synthesis of Cardiac Fibroblasts

**FIGURE 3:**
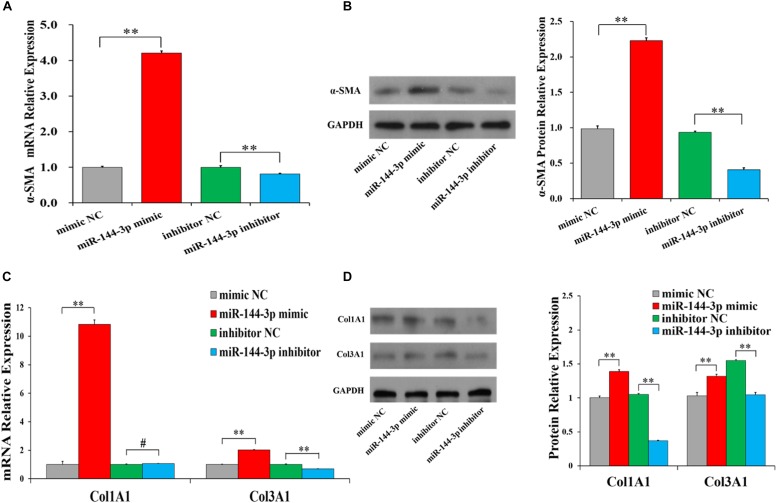
miR-144-3p accumulates the synthesis of ECM-related proteins in human cardiac fibroblasts (HCFs). The mRNA **(A)** and protein **(B)** expression of α*-SMA* in HCFs transfected with miR-144-3p mimics or miR-144-3p inhibitor. The mRNA **(C)** and protein **(D)** expression of *Col1A1* and *Col3A1* in HCFs transfected with miR-144-3p mimics or miR-144-3p. The protein expression was normalized by that of mimic NC group. The optical density of protein expression was shown in Supplementary Table S4. *N* ≥ 3. ^∗^*P* < 0.05, ^∗∗^*P* < 0.01, ^#^*P* > 0.05. Data are shown as mean ± SD.

To investigate the biological functions of miR-144-3p on collagen synthesis, the mRNA and protein levels of the ECM-related genes were detected with miR-144-3p mimic and inhibitor. We found that miR-144-3p mimic significantly increased the mRNA (Figure 3A) and protein (Figure 3B and Supplementary Table S4) levels of α*-SMA*, and miR-144-3p inhibitor could significantly decrease the mRNA (Figure 3A) and protein (Figure 3B) levels of α*-SMA* in HCFs. Moreover, miR-144-3p mimic was found to significantly increase the mRNA (Figure 3C) and protein (Figure 3D) levels of *Col3A1*, and miR-144-3p inhibitor could significantly decrease the mRNA (Figure 3C) and protein (Figure 3D) levels of *Col3A1* in HCFs. Besides, miR-144-3p mimic was observed to significantly increase the mRNA (Figure 3C) and protein (Figure 3D) levels of *Col1A1*, and miR-144-3p inhibitor shows no significant effect on the mRNA level (Figure 3C) but could significantly decrease the protein counts of *Col1A1* in HCFs (Figure 3D). These results demonstrated that miR-144-3p could accumulate the synthesis of ECM-related proteins, revealing that miR-144-3p might aggravate the cardiac fibrosis after MI.

### *PTEN* Is a Target of miR-144-3p

**FIGURE 4:**
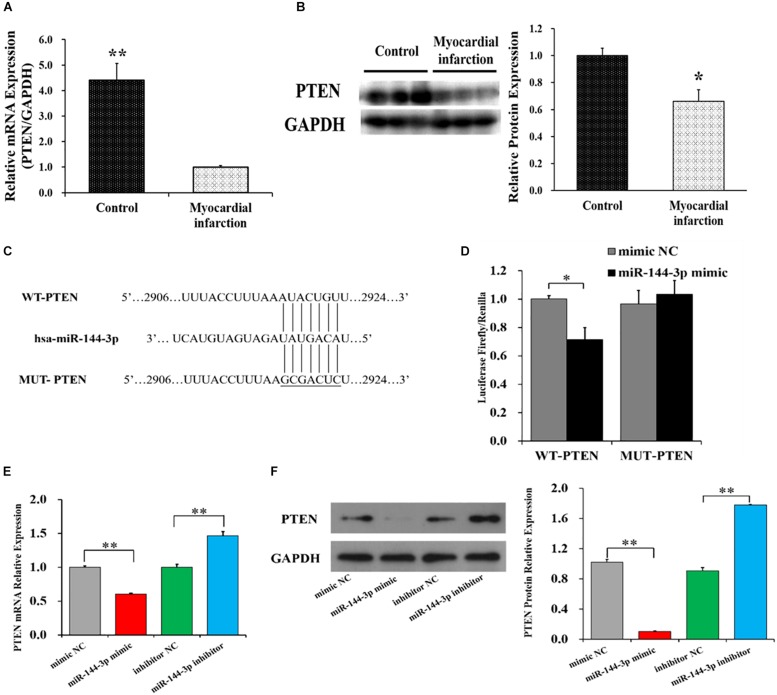
*PTEN* is a target of miR-144-3p. The mRNA **(A)** and protein **(B)** levels of PTEN in MI and control groups. **(C)** Wild-type sequence (WT-PTEN) and mutated sequence (MUT-PTEN) for miR-144-3p binding site. **(D)** Luciferase activities of WT-PTEN and MUT-PTEN. The mRNA **(E)** and protein **(F)** expression of *PTEN* in HCFs transfected with miR-144-3p mimics or miR-144-3p. The protein expression was normalized by that of mimic NC group **(F)**. The optical density of protein expression was shown in Supplementary Table S4. Data are shown as mean ± SD. *N* ≥ 3. ^∗^*P* < 0.05, ^∗∗^*P* < 0.01.

The putative target genes of miR-144-3p were predicted by three algorithms: TargetScan, MiRanda, and RNAhybrid. The phosphatase and tensin homology gene (*PTEN*), was predicted as the overlapped potential target gene by these three algorithms. To identify whether *PTEN* is a target of miR-144-3p, the mRNA and protein levels of *PTEN* were determined in MI and control groups (Figures 4A,B). The mRNA and protein levels of *PTEN* were observed to be negatively correlated with that of miR-144-3p (Figure 2A). To further validate *PTEN* as a target of miR-144-3p, the wild-type sequence (WT-PTEN) and mutated sequence (MUT-PTEN) of the putative miR-144-3p binding site of *PTEN*’s 3′UTR were cloned into the pmirGLO luciferase vector (Figure 4C). Compared to NC group, luciferase reporter assays showed that miR-144-3p mimics significantly decreased the luciferase activity of WT-PTEN but did not show a significant effect on the luciferase activity of MUT-PTEN (Figure 4D). Moreover, we found miR-144-3p mimic significantly down-regulated the mRNA (Figure 4E) and protein (Figure 4F and Supplementary Table S4) levels of *PTEN*, whereas miR-144-3p inhibitor up-regulated the mRNA (Figure 4E) and protein (Figure 4F and Supplementary Table S4) levels of *PTEN.* These results demonstrated that miR-144-3p targets the *PTEN*’s 3′UTR and repressed its expression at mRNA and protein levels.

### miR-144-3p Promotes the Proliferation, Migration, and Collagen Synthesis by Targeting *PTEN*

**FIGURE 5:**
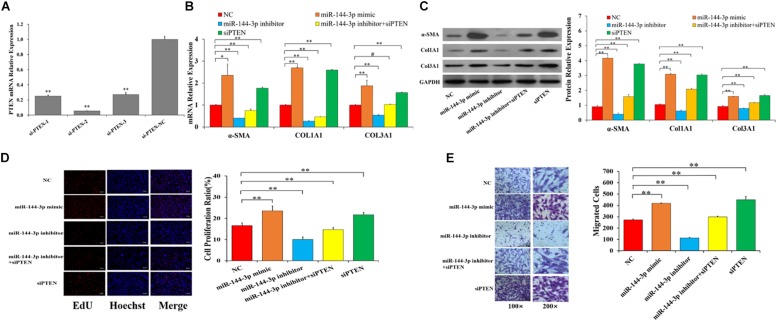
miR-144-3p promotes the proliferation, migration, and collagen synthesis by targeting *PTEN*. **(A)** The relative expression of *PTNE* knockdown by three siRNAs. miR-144-3p inhibitor reverses the siPTEN-mediated effects on the mRNAs **(B)** and protein **(C)** levels of α*-SMA* and *Col1A1*, and *Col3A1*. miR-144-3p inhibitor reverses the siPTEN-mediated effects on the cell proliferation **(D)** and migration **(E)** in HCFs. The protein expression was normalized by that of NC group. The optical density of protein expression was shown in Supplementary Table S5. Data are shown as mean ± SD. *N* ≥ 3. ^∗^*P* < 0.05. ^∗∗^*P* < 0.01. Bars: 100 μm for 100 × figures, and 50 μm for 200 × figures.

To explore whether miR-144-3p promoted myocardial fibrosis by directly targeting *PTEN* in HCFs, the expressions of *PTEN* was knocked down with specific siRNAs (Figure 5). Three PTEN-specific siRNAs (si-PTEN-1, si-PTEN-2, and si-PTEN-3) and a negative control (si-PTEN-NC) were transfected into HCFs. As shown in Figure 5A, si-PTEN-2 exhibited the best inhibition efficiency and thus was selected for knockdown of *PTEN* in HCFs. Compared with NC group, miR-144-3p mimics and siPTEN significantly increased the mRNA (Figure 5B) and protein (Figure 5C and Supplementary Table S5) expressions of α*-SMA* and *Col1A1*, and *Col3A1*, but miR-144-3p inhibitors and miR-144-3p inhibitors + siPTEN significantly decreased mRNA (Figure 5B) and protein (Figure 5C and Supplementary Table S5) expressions of α*-SMA* and *Col1A1*, and *Col3A1*. Moreover, EdU and transwell assays showed that miR-144-3p mimics and siPTEN significantly increased proliferation (Figure 5D) and migration (Figure 5E) of HCFs, but miR-144-3p inhibitors and miR-144-3p inhibitors + siPTEN significantly decreased proliferation (Figure 5D) and migration (Figure 5E) of HCFs, compared with NC group. These findings indicate that miR-144-3p inhibitor could reverse the siPTEN-mediated effects on fibrosis-related genes, proliferation and migration of HCFs, suggesting that miR-144-3p may directly target *PTEN* to participate in the occurrence of myocardial fibrosis.

## Discussion

Previous studies have suggested that pigs are a valuable biomedical model based the similarly genomic and physiological features to humans (Groenen et al., 2012; Madeja et al., 2019), and much evidence has indicated that pigs have been widely used as animal model in studies associated MI (Gabisonia et al., 2019; Lopez et al., 2019). In this study, the potential miRNAs that might involve in the processes of cardiac fibrosis after MI were identified in the miniature pigs. 84 miRNAs were identified as the expressed miRNAs (Figure 1 and Supplementary Table S3). Among these miRNAs, miR-21 (Cao et al., 2017), miR-29 (Zhu et al., 2013), miR-155 (Wei et al., 2017), miR-148 (Zhang et al., 2019), and miR-17 (Zhang et al., 2018) have been reported to participate in the processes of cardiac fibrosis, suggesting that the differentially expressed miRNAs identified in this study would provide useful information for the investigations on molecular mechanism for cardiac fibrosis after MI.

One previous study has demonstrated that the levels of miR-144-3p in serum was significantly associated with MI in humans (Hu et al., 2014), and miR-144-3p was predicted to involve in the cell-ECM interactions and in tissue remodeling (Floor et al., 2015). In humans, the circulating miR-144-3p was significantly associated with 10-year risk of MI and could significantly improve the risk prediction of MI (Bye et al., 2016; Velle-Forbord et al., 2019), and miR-144-3p inhibited the cardiomyocyte apoptosis after MI in mice (Gong et al., 2019). In this study, miR-144-3p was identified to be significantly higher expressed in MI area (Figures 1, 2A), compared to control pigs. Further investigations revealed that miR-144-3p could up-regulate the mRNAs and protein levels of the ECM-related genes, including α*-SMA*, *Col1A1*, and *Col3A1* (Figure 3), and promotes cell proliferation and migration of HCFs (Figure 2). These results suggested miR-144-3p might contribute the remodeling of ECM components and enhance the cardiac fibrosis after MI. Additionally, in our study, the detachment of cells was trypsinized during the cell migration experiment. The detachment by trypsin with ethylenediamine tetra-acetic acid will be better to measure cell migration (Fong et al., 2017).

In this study, miR-144-3p was observed to target at *PTEN*’s 3′UTR and repress its expression at mRNA and protein levels (Figure 4). Previous studies have recommended that *PTEN* suppresses the activation of PI3K-Akt signaling pathway (Maehama, 2006; Bassi and Mak, 2016) which has been suggested to accelerate MI (Liu S. et al., 2019) and regulated myocardial infarct size and fibrosis (Wang et al., 2019). Moreover, *PTEN* is reported to be down-regulated in myocardial tissue during the process of myocardial fibrosis after MI in rats (Zhang and Cui, 2018). In this study, we found that suppressing the expression of *PTEN* could up-regulate the mRNAs and proteins of ECM-related genes (α*-SMA*, *Col1A1*, and *Col3A1*) (Figures 5B,C) and promote the proliferation (Figure 5D) and migration (Figure 5E) of HCFs. These results are according with previous studies. For example, knockdown the expression of *PTEN* observably up-regulates the mRNA levels of *Col1A1* and *Col3A1* in mouse cardiac fibroblasts (Qu et al., 2019). During cardiac fibrosis of mice, the protein expression of PTEN is negatively correlated with that of α*-SMA* and *Col1A1* (Liu H.L. et al., 2019). Inhibition expression of *PTEN* promotes ECM deposition and cardiac fibrosis in mice (Zhang et al., 2018). These findings suggest that *PTEN* may alleviate the ECM deposition and cardiac fibrosis after MI.

It is well known that ECM provides mechanical support for the tissues and cellular phenotype (Shinde and Frangogiannis, 2014; Frangogiannis, 2017). Activated fibroblasts produce large amounts of ECM fibers (fibrillar collagens) in infarct area of heart after MI, and ECM fibers is likely to surround the migrating fibroblasts to provide “tracks” for cell movement (Dobaczewski et al., 2012; Frangogiannis, 2017), and excessive deposition of ECM fibers triggers cardiac fibrosis after MI (Zamilpa and Lindsey, 2010; Dobaczewski et al., 2012; van Nieuwenhoven and Turner, 2013; Lajiness and Conway, 2014; Tao et al., 2016; Frangogiannis, 2017). In the present study, taken together, miR-144-3p promotes cell proliferation, migration, and, collagen production by targeting *PTEN*, suggesting that miR-144-3p may be a new marker for cardiac fibrosis progression and that the miR-144-3p-mediated- PTEN regulation might be a novel therapeutic target for cardiac fibrosis after MI.

## Data Availability Statement

The small RNA sequencing data used in our study have been submitted on ENA under accession number: PRJEB34700 with URL: https://www.ebi.ac.uk/ena/data/view/PRJEB34700.

## Ethics Statement

The animal study was reviewed and approved by all experiments in the present study were performed in accordance with the guidelines of the Animal Care and Use Committee of Guangdong Provincial Key Laboratory for Laboratory Animals and Guangdong Laboratory Animals Monitoring Institute.

## Author Contributions

XY, HZ, and XW conceived and designed this work. XY, JP, BG, and HG acquired the biological samples and analyzed the data. LW, JP, and XY conducted the experiments and drafted the work. HZ, XW, JL, WT, and SL revised the draft critically. All authors reviewed and approved the final manuscript.

## Conflict of Interest

The authors declare that the research was conducted in the absence of any commercial or financial relationships that could be construed as a potential conflict of interest.

## Footnotes

1 https://microrna-discovery-by-deep-sequencing.soft112.com/

2 http://www.mirbase.org

3 http://www.targetscan.org/

4 http://mirdb.org/

5 https://bibiserv.cebitec.uni-bielefeld.de/rnahybrid/

6 http://www.webgestalt.org/2017/option.php
