# Supplementary material for: MiR-144-3p Enhances Cardiac Fibrosis After Myocardial Infarction by Targeting PTEN
Source: Front Cell Dev Biol. 2019 Oct 29;7:249. doi: 10.3389/fcell.2019.00249 (PMC6828614; doi:10.3389/fcell.2019.00249)
Supplement: Supplementary file 1 [file Table_1.DOCX]

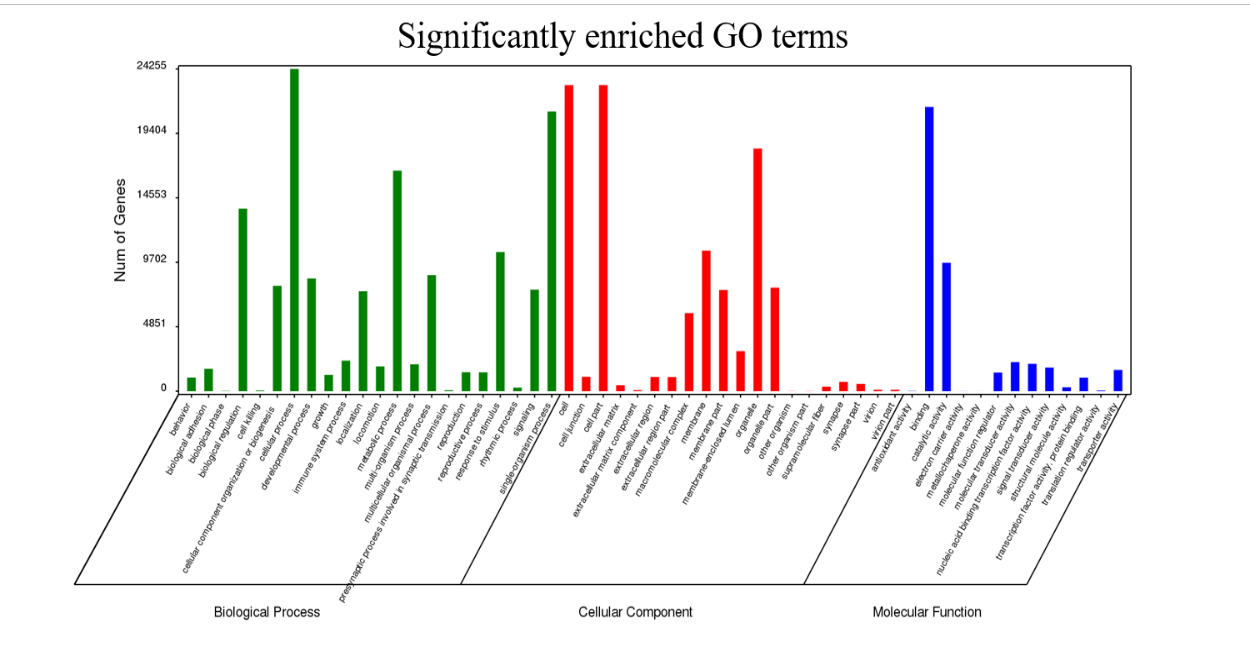


Figure S1. GO terms of potential target genes of these differentially expressed miRNAs.


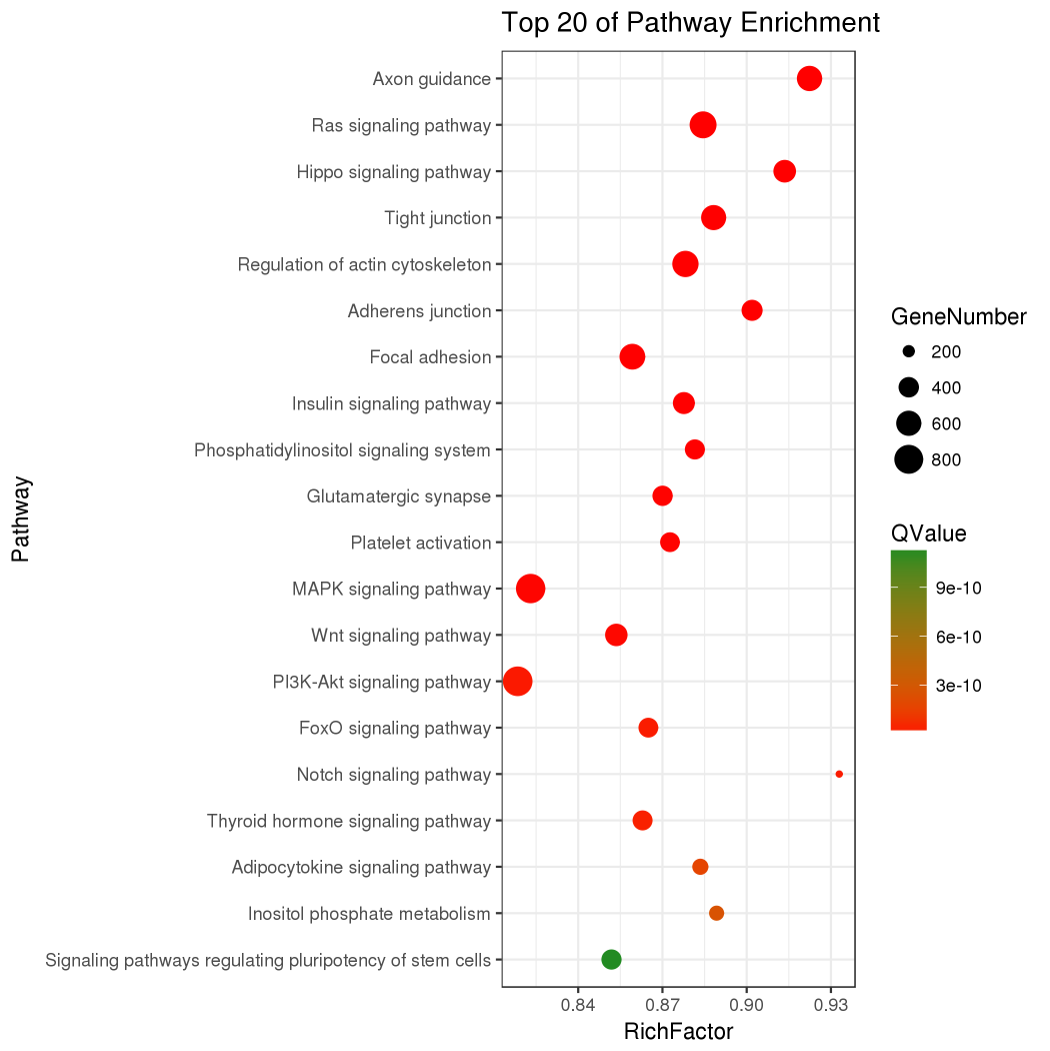


Figure S2. KEGG analysis of potential target genes of these differentially expressed miRNAs.
